# Risk of dementia from proton pump inhibitor use in Asian population: A nationwide cohort study in Taiwan

**DOI:** 10.1371/journal.pone.0171006

**Published:** 2017-02-15

**Authors:** Shu-Yu Tai, Chen-Yu Chien, Deng-Chyang Wu, Kun-Der Lin, Bo-Lin Ho, Yu-Han Chang, Yang-Pei Chang

**Affiliations:** 1 Department of Family Medicine, School of Medicine, College of Medicine, Kaohsiung Medical University, Kaohsiung City, Taiwan; 2 Department of Family Medicine, Kaohsiung Medical University Hospital, Kaohsiung Medical University, Kaohsiung City, Taiwan; 3 Department of Family Medicine, Kaohsiung Municipal Ta-Tung Hospital, Kaohsiung Medical University Hospital, Kaohsiung Medical University, Kaohsiung City, Taiwan; 4 Department of Otorhinolaryngology, School of Medicine, College of Medicine, Kaohsiung Medical University, Kaohsiung City, Taiwan; 5 Department of Otorhinolaryngology, Kaohsiung Medical University Hospital, Kaohsiung Medical University, Kaohsiung City, Taiwan; 6 Department of Otorhinolaryngology, Kaohsiung Municipal Hsiao-Kang Hospital, Kaohsiung Medical University, Kaohsiung City, Taiwan; 7 Division of Gastroenterology, Department of Internal Medicine, Kaohsiung Medical University Hospital, Kaohsiung City, Taiwan; 8 Department of Internal Medicine, Kaohsiung Municipal Ta-Tung Hospital, Kaohsiung City, Taiwan; 9 Center for Stem Cell Research, Kaohsiung Medical University, Kaohsiung City, Taiwan; 10 Division of Endocrinology and Metabolism, Department of Internal Medicine, Kaohsiung Medical University Hospital, Kaohsiung City, Taiwan; 11 Division of Endocrinology and Metabolism, Kaohsiung Municipal Ta-Tung Hospital, Kaohsiung Medical University Hospital, Kaohsiung City, Taiwan; 12 Department of Neurology, Kaohsiung Municipal Ta-Tung Hospital, Kaohsiung Medical University, Kaohsiung City, Taiwan; 13 Department of Neurology, Kaohsiung Medical University Hospital, Kaohsiung Medical University, Kaohsiung City, Taiwan; 14 Management Office, Kaohsiung Municipal Ta-Tung Hospital, Kaohsiung Medical University, Kaohsiung City, Taiwan; Istituto Di Ricerche Farmacologiche Mario Negri, ITALY

## Abstract

**Introduction:**

Concerns have been raised regarding the potential association between proton pump inhibitor (PPI) use and dementia.

**Objective:**

This study aimed to examine this association in an Asian population.

**Methods:**

Patients initiating PPI therapy between January 1, 2000 and December 31, 2003 without a prior history of dementia were identified from Taiwan’s National Health Insurance Research Database. The outcome of interest was all-cause dementia. Cox regression models were applied to estimate the hazard ratio (HR) of dementia. The cumulative PPI dosage stratified by quartiles of defined daily doses and adjusted for baseline disease risk score served as the primary variables compared against no PPI use.

**Results:**

We analyzed the data of 15726 participants aged 40 years or older and free of dementia at baseline. PPI users (n = 7863; average follow-up 8.44 years) had a significantly increased risk of dementia over non—PPI users (n = 7863; average follow-up 9.55 years) (adjusted HR [aHR] 1.22; 95% confidence interval: 1.05–1.42). A significant association was observed between cumulative PPI use and risk of dementia (*P* for trend = .013). Subgroup analysis showed excess frequency of dementia in PPI users diagnosed with depression (aHR 2.73 [1.91–3.89]), hyperlipidemia (aHR 1.81 [1.38–2.38]), ischemic heart disease (aHR 1.55 [1.12–2.14]), and hypertension (aHR 1.54 [1.21–1.95]).

**Conclusions:**

An increased risk for dementia was identified among the Asian PPI users. Cumulative PPI use was significantly associated with dementia. Further investigation into the possible biological mechanisms underlying the relationship between dementia and PPI use is warranted.

## Introduction

Dementia is a chronic, progressive, multifactorial neurodegenerative disorder characterized by a decline in cognitive function. With the increase in the aging population, the World Health Organization estimates the proportion of dementia cases in people aged 60 years and older will reach 22% worldwide by 2050[1], with Asia estimated to account for 59% of the cases worldwide[2]. The consequent high demand for medical therapy and care needed to treat cumulative cognitive decline will have considerable socioeconomic impact. The estimated worldwide costs of treating dementia were estimated to be US$604 billion in 2010[3]. Thus, the prevention of dementia in populations at increased risk (e.g., the elderly) may help reduce the burden caused by dementia on people and healthcare systems. Therefore, it is no surprise that commonly used drugs that could potentially increase or decrease the risk of dementia in the elderly as a consequence of their long-term use have been examined in epidemiological studies.

Evidence suggests that the precipitation of β-amyloid (Aβ) peptide in the central nervous system can lead to the development of dementia[4]. Proton pump inhibitors (PPIs), which act as remarkable and long-lasting reducers of gastric acid production, are prescribed for the treatment for acid-related conditions such as gastroesophageal reflux disease and peptic ulcers[5, 6]. Their use has increased, especially among the elderly[7, 8]. PPI use might lower cognition by enhancing Aβ levels in the brains of mice by affecting the enzymes β- and γ-secretases[9] or by modulating the degradation of Aβ by lysosomes in microglia[10–13]. Lam et al[14] reported a significant association of previous and current PPI use with vitamin B12 deficiency in a population-based sample. Vitamin B12 deficiency has been associated with cognitive decline [15]. A prospective, longitudinal, multicenter cohort study of elderly primary care patients in Germany, including 3327 community-dwelling persons aged 75 years or older, found a significant association between PPI use and incident dementia (hazard ratio [HR], 1.38 [95%CI, 1.04–1.83])[13]. Another prospective cohort study, derived from data supplied by the largest German statutory health insurer, reported that avoiding the use PPI may reduce the risk of dementia[16]. These studies, mostly based on western populations, show increased interest in whether PPIs can increase the incidence and progression of dementia. In this study, we investigated potential association between proton pump inhibitor (PPI) use and dementia in an Asian population using Taiwan’s National Health Insurance Research Database (NHIRD) to follow the development of dementia in users and non-users of PPIs in a Taiwanese population over a >10-year period (1997–2010).

## Methods

### Data source

The present study was conducted using claims data from the National Health Insurance Research Database (NHIRD), which is managed by the National Health Research Institute (NHRI) in Taiwan. Taiwan’s National Health Insurance (NHI) provides reimbursements for healthcare costs for 99% of the population in Taiwan (approximately 23 million people). The NHIRD contains comprehensive healthcare information, including demographic data of insured individuals, dates of clinical visits, diagnostic codes, and prescription details. The data of this study was obtained from the Longitudinal Health Insurance Database (LHID) 2000, a subset of the NHIRD. The LHID 2000 dataset contains historical ambulatory and inpatient care data for one million randomly sampled beneficiaries enrolled in the NHI system in 2000. The LHID 2000 database allows researchers to follow the medical service utilization history of these patients. The claims found in the LHID 2000 and NHIRD do not differ significantly in age, sex, or healthcare costs.

### Study patients

**Fig 1:**
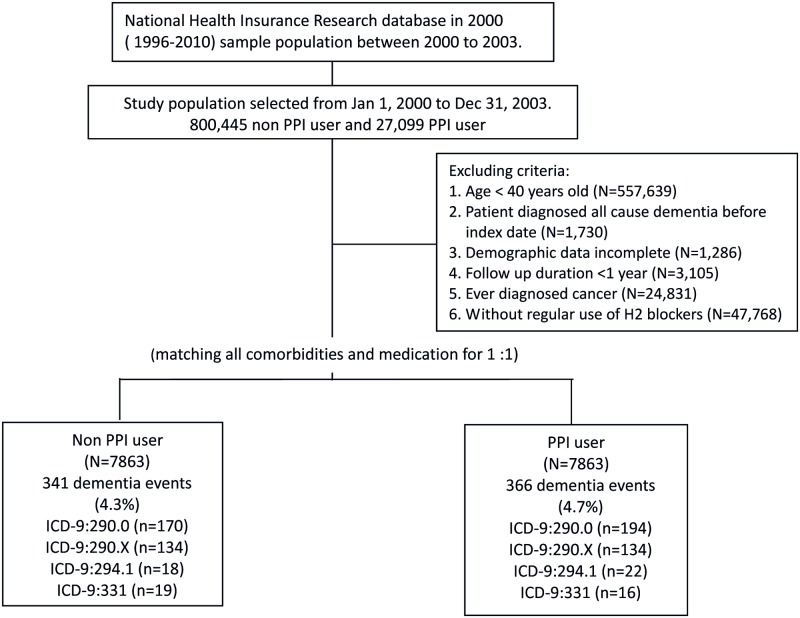
Study flowchart.

Patients who had ever received PPI between January 1, 2000 and December 31, 2003, were identified for the PPI cohort (PPI users) and were compared with a comparison cohort comprised of patients who had never been treated with PPI (hereafter, non—PPI users); these patients were randomly sampled from the remaining enrollees of the LHID 2000 data set and were 1:1 matched for age, sex, propensity score, and index year. The date of initial use of PPI for each patient was assigned as their index date. Initiation was defined as being free from any PPI therapy for 12 months prior to the first prescription (index date). Patients were excluded if they were less than 40 years of age (N = 557 639), had all-cause dementia diagnosed before the index date (N = 1730), had incomplete demographic data (N = 3105), had follow-up durations less than 1 year, were ever diagnosed with cancer (N = 24 831), or currently/previously regularly used H2 blockers (more than 3 months in 1 year) (N = 47 768) (Fig 1).

### Cumulative exposure of PPI

Drug use information was obtained from the outpatient pharmacy prescription database. It included prescribed drug dosage, date of prescription, supply days, and total number of pills dispensed. Because patients might discontinue or restart drug therapy, we assumed that patients’ exposure to each studied drug contributed both cumulatively and continuously to their long-term risk of dementia. Defined daily dose (DDD) is the assumed average maintenance dose per day for a drug used for its main indication in adults[17]. To investigate the effect of dose, the cumulative use of PPI was calculated as total prescribed DDD (i.e., the same as total dispensed DDD under this system) analysis. When a dementia event occurred, the cumulative PPI dosage was recorded as a total of DDD from drug initiation to the day that the dementia event occurred. For those who were still at risk (event free and uncensored), the cumulative doses were recorded and ranked at each event time. Participants were then classified into mutually exclusive dosage categories on the basis of quartiles of cumulative dosage distribution of the “risk set” at that time[18]. As the PPI dosage accumulated during the follow-up period, a participant could be reassigned to either a higher or a lower quartile.

### Ascertainment of dementia

The outcome of interest was defined as a diagnosis of ICD-9-CM: 290.0 (senile dementia, uncomplicated), 290.4x (arteriosclerotic dementia), 294.1 (dementia in conditions classified elsewhere), and 331.0 (Alzheimer disease). Patients diagnosed with dementia were required to have at least two outpatient visits or one inpatient hospitalization for dementia, and a diagnosis made by a neurologist or psychiatrist. Patients were followed from the index date to the earliest outcome of occurrence, death, and disenrollment from the NHI or the end of the study date (December 31, 2010), whichever occurred first. This study was approved by the Institutional Review Board of Kaohsiung Medical University Hospital (KMUHIRB-EXEMPT (II)-20160028). Because the patient identifiers are scrambled to researchers to protect patient confidentiality, the requirement for written or verbal consent from patients for data linkage study was waived.

### Covariate ascertainment and adjustment

Inpatient and outpatient files from the year prior to the index date were used to ascertain whether they had comorbidities, including diabetes mellitus (ICD-9-CM: 250.xx), hypertension (ICD-9-CM: 401x), hyperlipidemia (ICD-9-CM: 272x), peripheral vascular disease (ICD-9-CM: 443), ischemic heart disease (ICD-9-CM: 410.x-414.x), depression (ICD-9-CM: 296.x-, 300.4, 311), and ischemic stroke (ICD-9-CM: 433–438) as well as the Charlson’s comorbidity index(CCI)[19]. We categorized CCI into 3 levels ≦1, 2, and ≧3. CCI was determined for each subject from claims data for outpatient visits or hospitalizations at baseline. The CCI is a scoring system that includes weighting factors on important concomitant diseases; it has been validated for the use with ICD-9-CM coded administrative database [20]. Demographic data, including age, sex, and urbanization, were extracted. Comorbidities were defined in a patient if he or she was diagnosed for any of the aforementioned diseases on at least two outpatient claims or one inpatient claim during the exposure period. We included drugs as confounding agents if they could potentially accelerate or reduce inflammation or cognitive function in the model. These included anticoagulants, nonsteroidal anti-inflammatory agents (NSAIDs), antiplatelet agents, antidiabetic agents, antihypertensives, and statins. Exposure to these drugs was defined as having a prescription for one of them from at least one day after the index date to the occurrence of any event related to this study, being disenrolled from the NHI program, death, or the end of the study period (December 31, 2010), whichever occurred first.

### Statistical analysis

Pearson chi-square test was used to evaluate differences in categorical data between PPI users and non—PPI users, including demographic data and comorbidities. Cox hazards regression analysis was performed to examine the risk of dementia among PPI users compared with non—PPI users during the follow-up period. The DDD recommended by WHO was used to quantify the use of PPI. Cumulative DDD was estimated as the sum of dispensed DDDs of PPI from January 1, 2000 to the data of a diagnosis of dementia or until the end of the study. PPI users were categorized into users of extremely low doses (<28 DDDs), low doses (28–48 DDDs), moderate doses (49–83 DDDs), and high doses (>84 DDDs). Several covariables such as age, gender, urbanization, Charlson’s index, and all comorbidities and comedications were adopted in the statistical analysis model. Hazard ratios (HRs) and 95% confidence intervals (CIs), using the comparison cohort of non—PPI users as the reference, were calculated to show the risk of dementia in PPI users and in the dose—response analysis of cumulative PPI use. Subgroup analysis was done restricted to aged older than 60 y/o patients, who were more likely to develop dementia. Data analysis was performed using the SAS 9.3 statistical package; all *P*-values were 2-sided, and *P* < .05 was considered significant.

## Results

**Fig 2:**
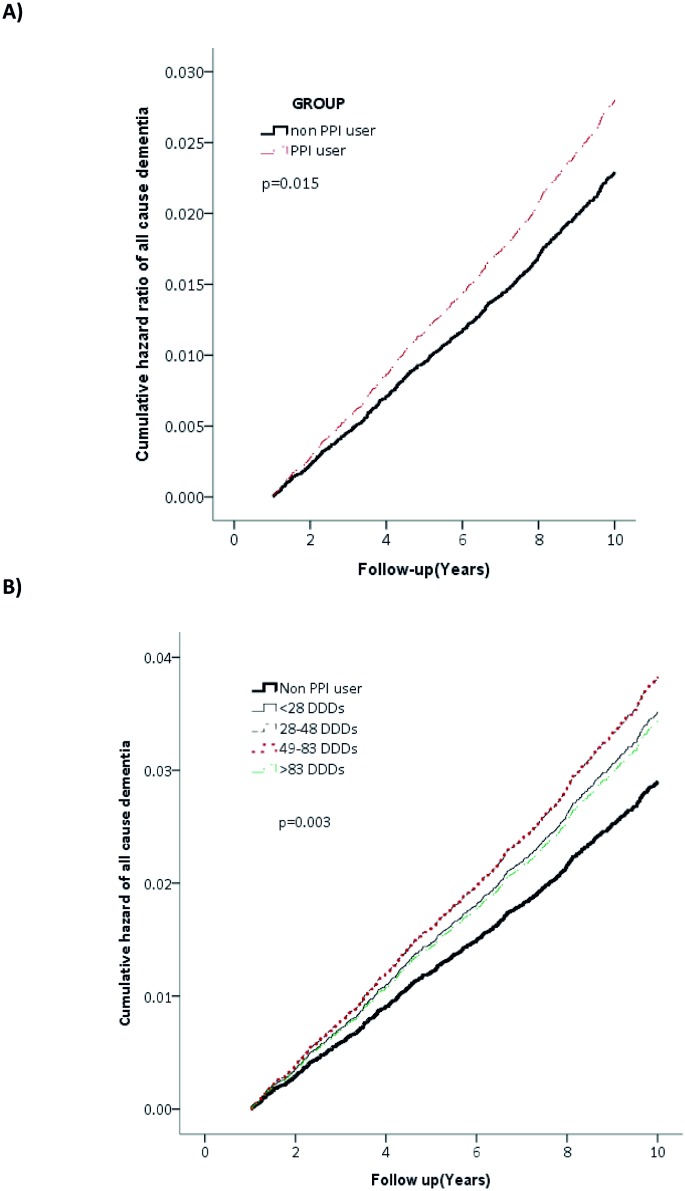
All-cause dementia by A) use and B) defined daily dose (DDD) of proton pump inhibitors (PPIs).

A total of 7863 PPI initiators from January 1, 2000, to December 31, 2003, were identified (Fig 1). After propensity score matching, 7863 PPI users were matched to the 7863 non—PPI users for the final analysis. The characteristics of both groups are listed in Table 1. All covariates were comparable after matching (Table 1). Among the study participants, 707 all-cause dementia cases occurred with an average follow-up of 9.0 years. The crude incidence rate was 5.51 per 1000 person-years among PPI users and 4.54 among non—PPI users. PPI users had a slightly higher risk of developing dementia than non-PPI users after adjusting for the covariates listed in Table 1. (HR, 1.22 [95%CI, 1.05–1.42]; Table 2) Furthermore, we found a significant association between cumulative PPI use and all-cause dementia (*P* for trend = .013; Table 2). The findings were similar among patients of inclusion age older than 60 y/o. (Table A in S1 File) The survival curve of the PPI use and incident dementia is depicted in Fig 2. Fig 2 depicts all-cause dementia by (A) use and (B) defined daily dose (DDD) of proton pump inhibitors (PPIs).

**Table 1:** Basic characteristics of PPI users and non—PPI users (N = 15,726).

Variables	Non PPI user (N = 7863)		PPI user (N = 7863)		P value
	N	(%)	N	(%)	
Age					
40–49	3316	(42.2)	3168	(40.3)	0.186
50–59	1912	(24.3)	2046	(26.0)	
60–69	1364	(17.3)	1356	(17.2)	
≧70	1271	(16.2)	1293	(16.4)	
Mean (±SD)	55.33	(±12.23)	55.65	(±12.37)	0.102
Gender					
Female	3199	(40.7)	3238	(41.2)	0.527
Male	4664	(59.3)	4625	(58.8)	
Urbanization					
Urban & Suburban	5880	(74.8)	5811	(73.9)	0.208
Rural	1983	(25.2)	2052	(26.1)	
Comorbidities					
Diabetes	862	(11.0)	814	(10.4)	0.215
Hyperlipidemia	1035	(13.2)	1020	(13.0)	0.723
hypertension	1925	(24.5)	1924	(24.5)	0.985
Depression	391	(5.0)	370	(4.7)	0.435
Ischemic heart disease	314	(4.0)	306	(3.9)	0.743
Cerebral vascular disease	172	(2.2)	184	(2.3)	0.520
CCI score					
≦1	6796	(86.4)	6795	(86.4)	0.112
2	840	(10.7)	800	(10.2)	
≧3	227	(2.9)	268	(3.4)	
Medication					
Anticoagulant agents	35	(0.4)	33	(0.4)	0.808
Antiplatete agents	233	(3.0)	234	(3.0)	0.963
Antidiabetic agents	549	(7.0)	539	(6.9)	0.753
Antihypertension agents	1465	(18.6)	1434	(18.2)	0.524
statin	184	(2.3)	171	(2.2)	0.485
NSAIDs	586	(7.5)	601	(7.6)	0.651

PPI: proton pump inhibitor

SD: standard deviation

NSAIDs: nonsteroidal anti-inflammatory agents

CCI score: Charlson’s comorbidity index score

**Table 2:** Risk of all-cause dementia among non—PPI users and PPI users (N = 15,726).

	No. cases	Per 1,000 Person year	cHR	(95%CI)	p value	aHR	(95%CI)	p value
Non PPI user	341	4.54	Ref.			Ref.		
PPI user	366	5.51	1.25	(1.08–1.46)	0.003	1.22	(1.05–1.42)	0.009
Cumulative DDDs								
<28	72	4.38	0.99	(0.77–1.28)	0.962	1.21	(0.94–1.56)	0.153
28–48	83	5.15	1.17	(0.92–1.49)	0.209	1.32	(1.04–1.68)	0.023
49–83	108	5.85	1.33	(1.07–1.65)	0.011	1.33	(1.07–1.65)	0.011
≧84	103	6.73	1.54	(1.23–1.92)	<0.001	1.19	(0.95–1.48)	0.133
p for trend					<0.001			0.013

Adjusted for age, gender, urbanization, all comorbidities, CCI score, and medication.

PPI: proton pump inhibitor

cHR: crude hazard ratio

aHR: adjusted hazard ratio

DDD: defined daily dose

CCI score: Charlson’s comorbidity index score

A subgroup analysis was performed for the most used PPI subtypes (omeprazole, pantoprazole, and lansoprazole) in Taiwan during 2000–2003. After adjusting for covariates, we found a similar elevated risk of dementia for omeprazole (HR, 1.30 [95%CI, 1.09–1.54]) and similar but not significantly elevated risk for pantoprazole (HR, 1.36 [95%CI, 0.98–1.89]) and lansoprazole (HR, 1.20 [95%CI, 0.98–1.46]). (Table B in S1 File) The survival curve of subtype PPI use and incident dementia is presented in Fig A of S1 File.

Male PPI users had significantly higher risk of developing dementia (HR, 1.24 [95%CI, 1.01–1.52]), whereas female users did not (HR, 1.21 [95%CI, 0.97–1.50]), compared with their non—PPI user counterparts. (Table C in S1 File)

Compared with non—PPI users, PPI users without diabetes mellitus (HR, 1.29 [95%CI, 1.09–1.52]), depression (HR, 1.20 [95%CI, 1.02–1.40]), or cerebral vascular disease (HR, 1.25 [95%CI, 1.07–1.46]) and those with hyperlipidemia (HR, 1.49 [95%CI, 1.06–2.10]) and ischemic heart disease (HR, 2.18 [95%CI, 1.31–3.63]) had significantly higher risk of developing dementia. PPI users not taking anticoagulant agents (HR, 1.23 [95%CI, 1.06–1.43]), antiplatelet agents (HR, 1.20 [95%CI, 1.02–1.40]), antidiabetic agents (HR, 1.28 [95%CI, 1.09–1.51]), statins (HR, 1.18 [95%CI, 1.03–1.40]), or NSAIDs (HR, 1.20 [95%CI, 1.02–1.41]) had significantly higher risk of developing dementia than non—PPI users. In contrast, PPI users taking antihypertension agents (HR, 1.46 [95%CI, 1.14–1.85]) had significantly higher risk of developing dementia than non—PPI users. (Table C in S1 File)

Table 3 shows the additive effect of PPI use and covariates. Depression (aHR, 2.73 [95%CI, 1.91–3.89]) and hyperlipidemia (aHR, 1.81 [95%CI, 1.38–2.38]) were associated with the highest increased risk for incident dementia. In addition, being more than 70 years old was also associated with increased risk of dementia (aHR, 1.38 [95%CI, 1.02–1.87]). Other comorbidities were also significantly associated with elevated the risk of dementia. They were hypertension (aHR, 1.54 [95%CI, 1.21–1.95] and ischemic heart disease (aHR, 1.55 [95%CI, 1.12–2.14]). In addition, PPI use along with the following comedications had a considerable effect on risk of dementia: antiplatelet agents (aHR, 1.91 [95%CI, 1.37–2.66]), antihypertensives (aHR, 1.36 [95%CI, 1.07–1.38]), statins (aHR, 1.59 [95%CI, 1.00–2.53]), and NSAIDs (aHR, 1.19 [95%CI, 1.21–2.09])

**Table 3:** The additive effects of covariates on all cause dementia between non PPI user and PPI user (N = 15,726).

	Non PPI user		PPI user	
	aHR (95% CI)	p value	aHR (95% CI)	p value
Age				
<70	Ref.		1.11(0.88–1.40)	0.375
≧70	1.06(0.79–1.43)	0.707	1.38(1.02–1.87)	0.035
Comorbidites				
Hyperlipidemia				
No	Ref.		1.18(0.99–1.39)	0.053
Yes	1.29(0.97–1.71)	0.082	1.81(1.38–2.38)	<0.001
Hypertension				
No	Ref.		1.19(0.96–1.46)	0.109
Yes	1.22(0.96–1.55)	0.106	1.54(1.21–1.95)	<0.001
Depression				
No	Ref.		1.19(1.02–1.40)	0.028
Yes	1.78(1.24–2.56)	0.002	2.73(1.91–3.89)	<0.001
Ischemic heart disease				
No	Ref.		1.51(0.98–1.34)	0.080
Yes	0.71(0.47–1.08)	0.113	1.55(1.12–2.14)	0.008
Medication				
Antiplatete agents				
No	Ref.		1.19(1.01–1.39)	0.035
Yes	1.19(0.80–1.75)	0.389	1.91(1.37–2.66)	<0.001
Antihypertension agents				
No	Ref.		1.10(0.91–1.33)	0.346
Yes	0.94(0.73–1.22)	0.648	1.36(1.07–1.38)	0.014
Statin				
No	Ref.		1.20(1.03–1.40)	0.021
Yes	0.91(0.52–1.59)	0.741	1.59(1.00–2.53)	0.049
NSAIDs				
No	Ref.		1.19(1.01–1.41)	0.034
Yes	1.15(0.86–1.55)	0.349	1.19(1.21–2.09)	0.001

Adjusted for age, gender, urbanization, all comorbidities, CCI score, and medication.

PPI: proton pump inhibitor

aHR: adjusted hazard ratio

NSAIDs: non-steroidal anti-inflammatory agents

CCI score: Charlson’s comorbidity index score

## Discussion

This study based on claims data made available by Taiwan’s National Insurance Research Database (NHIRD) revealed a significant increased risk of dementia associated with the use of PPIs in an Asian population, confirming the findings of two German studies using AgeCoDe and AOK datasets[16, 21].

PPIs have been used since the early nineties for the treatment of gastrointestinal disorders associated with excessive synthesis of gastric acid[22]. This class of drugs exerts a stronger acid-suppressing effect than other traditional therapies, e.g., histamine-2(H2) receptor antagonists[22]. PPI use has increased significantly over the last 15 years, particularly in elderly populations [7, 8]. Despite initial reports of the safety of PPI use, some recent epidemiological studies have reported adverse effects with their use, including short-term effects (e.g., *Clostridium difficile* infection and pneumonia[23]) and/or long-term effects (e.g., osteoporosis, hip fractures, nutritional deficiencies [24], the risk of cardiovascular morbidity and mortality [25, 26], and renal failure[27]). Although these epidemiological studies are limited in their design, there is a need to be concerned about these complications among older patients, who are at particularly high risk.

The pharmacoepidemiological results of the current Asian study and the recent two German studies indicate an association between PPI and dementia. The exact mechanism though which PPIs might influence the development of dementia is still unknown. However, evidence indicates that PPIs first cross the blood—brain barrier[28] and interact with certain brain enzymes that could increase Aβ production[9] in the brain and decreasing its degradation[13]. PPIs may also bind to tau protein[28]. Thus, although Aβ is considered the most relevant factor related to the development of dementia, tau protein probably plays a role as well [28]. Another possible mechanism of PPI-induced dementia could be vitamin B-12 deficiency resulting from chronic PPI use[14]. Deficiency of this essential compound has been associated with cognitive decline [29, 30]. Furthermore, some evidence indicates that chronic exposure of human endothelial cells to PPIs accelerates endothelial aging, leading to dementia[31].

Our results are comparable to those based on AOK data, which was a claims data analysis adjusted for almost all of the potential confounding factors[16]. but not to the AgeCoDe study [21], which included the ApoE4 allele status and educational level. We found PPI users to be clearly at significant elevated risk for developing dementia compared with non—PPI users regardless of age, sex, and comorbidity or comedication. Furthermore, increased risk of dementia was noted in the presence of comorbidities of hyperlipidemia, hypertension, depression, and ischemic heart disease. Numerous studies have suggested a link between depression and dementia [32–36]. In our study, PPI users with depression were also found to have a higher risk for developing dementia. Furthermore, PPI users also had higher risk of developing dementia with concomitant use of antiplatelet agents or statins compared with other comorbidities and comedications. This might be explained by poor vascular status.

Our subgroup analyses for the three most used PPIs (omeprazole, pantoprazole, and lansoprazole) during 2000–2003 in Taiwan showed similar effect sizes, though there was only significantly pronounced risk of dementia associated with the use of omeprazole. Omeprazole is the first PPI to be approved (in 1988); at that time, it was found to have faster, stronger, and longer acid reduction than the histamine H_2_ receptor antagonists. Subsequently, lansoprazole (1991), pantoprazole (1994), rabeprazole (1999), and esomeprazole (2001) were approved [37]. PPIs have become a mainstream treatment of gastric acid-related diseases as well as a first-line medication[38]. In Taiwan, the time that pharmaceuticals are approved and listed is usually 2–3 years later than the global listing time, so the most popular PPIs during the recruiting period (2000–2003) were omeprazole, pantoprazole, and lansoprazole, which are different from the Germany claims data (omeprazole, pantoprazole, and esomeprazole)[16]. PPIs have dramatically improved the management options available for patients with acid-related disorders. Most patients with gastroesophageal reflux disease have excellent outcomes when initially prescribed PPIs, though some patients may need to switch to a different PPI during treatment. In our subgroup analysis, we found PPI rotations to be popular in clinical practice in Taiwan. We tried to reduce this confounding factor by adjusting for the other subtype PPI use.

The proportion of elderly people in Taiwan has risen over the past three decades from 4.1% in 1980 to 11.5% in 2013, the highest rate of aging worldwide [39]. Dementia will, therefore, be a major public health problem Taiwan. Another problem in the elderly population is polypharmacy. Some drugs may lead to injury of gastric mucosa (e.g., aspirin and NSAID), which, in turn, lead to tremendously increased use of PPIs among the elderly [7, 8]. This is a crucial issue given the very high prevalence of polypharmacy and long-term use among elderly populations who are already at greater risk of dementia.

The strength of this study is that it is a national cohort study based on Taiwan’s NHIRD, which contains data From Taiwan’s compulsory and universal healthcare system which has high coverage rate in Taiwan. This allowed us to perform our analysis in a real-life setting in an unselected patient population. In addition, patient dropout was avoided and selection bias or recall bias minimized because of the use of routine database records.

The study also has several limitations. One is that it was a retrospective review of the medical records and did not include results from formal cognitive function testing. Another limitation is that Alzheimer’s disease (AD) and vascular dementia often coexist as a mixed dementia. We could not differentiate between different dementia etiologies using the NHIRD because there is no distinguishing information available from the diagnostic codes of ICD-9-cm. This fact may not be a major limitation since the AgeCoDe study [21] found that there was only subtle differences between all dementia patients and AD patients and mixed dementia in most dementia cases [40, 41]. The diagnoses of dementia in NHIRD were based on ICD-9-CM codes and were determined by relevant specialists and physicians, according to standard clinical criteria. The data on the diagnoses of dementia can thus be considered reliable. However, the degree or stage of dementia was not available in this claims data. Still another limitation is that the NHIRD lacks detailed information on smoking habits, dietary preference, occupational exposure, educational level and socioeconomic status, all of which are potential risk factors for dementia. Moreover, all data in the NHIRD are anonymous; therefore, relevant clinical variables such as serum laboratory data, genetic factors (e.g., ApoE4 allele carrier), imaging and pathology results were also unavailable. Finally, although the findings of this study can be generalized to the Taiwanese population, applicability to other ethnicities should be revalidated.

## Conclusions

This population-based cohort study found Asian PPI users to be at increased risk for dementia and that cumulative PPI use was significantly associated with dementia. This finding should be further examined through randomized, prospective clinical trials.

## Supporting information

S1 File Relevant data underlying the findings described in manuscript. (DOC)
