# Supplementary material for: Risk of dementia from proton pump inhibitor use in Asian population: A nationwide cohort study in Taiwan
Source: PLoS One. 2017 Feb 15;12(2):e0171006. doi: 10.1371/journal.pone.0171006 (PMC5310771; doi:10.1371/journal.pone.0171006)
Supplement: S1 File — (DOC) [file pone.0171006.s001.doc]

**Table A. The risk of all cause dementia between non-PPI users and PPI users among different age group (N=15,726)**

|  | No. cases | Per 1,000  Person year |  | cHR | (95%CI) | p value | aHR | (95%CI) | p value |
| --- | --- | --- | --- | --- | --- | --- | --- | --- | --- |
| Age 40-49 years old | (N=6484) | |  |  |  |  |  |  |  |
| Non PPI user | 18 | 0.13 |  | Ref. |  |  | Ref. |  |  |
| PPI user | 13 | 0.47 |  | 1.06 | (0.51-2.20) | 0.872 | 1.05 | (0.50-2.17) | 0.904 |
| Cumulative DDDs |  |  |  |  |  |  |  |  |  |
| <21 | 4 | 0.53 |  | 1.19 | (0.40-3.57) | 0.751 | 1.20 | (0.40-3.58) | 0.745 |
| 21-41 | 2 | 0.31 |  | 0.70 | (0.16-3.03) | 0.630 | 0.73 | (0.17-3.16) | 0.671 |
| 42-72 | 3 | 0.44 |  | 0.99 | (0.29-3.38) | 0.981 | 0.96 | (0.28-3.28) | 0.946 |
| ≧73 | 4 | 0.58 |  | 1.35 | (0.45-4.03) | 0.595 | 1.25 | (0.42-3.75) | 0.692 |
| p for trend |  |  |  |  |  | 0.789 |  |  | 0.790 |
| Age 50-59 years old | (N=3958) | |  |  |  |  |  |  |  |
| Non PPI user | 32 | 1.72 |  | Ref. |  |  | Ref. |  |  |
| PPI user | 38 | 2.17 |  | 1.28 | (0.80-2.07) | 0.305 | 1.30 | (0.81-2.10) | 0.280 |
| Cumulative DDDs |  |  |  |  |  |  |  |  |  |
| <28 | 12 | 2.04 |  | 1.21 | (0.62-2.35) | 0.584 | 1.23 | (0.63-2.41) | 0.539 |
| 28-48 | 7 | 2.42 |  | 1.43 | (0.63-3.25) | 0.393 | 1.42 | (0.63-3.24) | 0.399 |
| 49-83 | 7 | 1.67 |  | 0.99 | (0.43-2.25) | 0.976 | 1.04 | (0.46-2.36) | 0.931 |
| ≧84 | 12 | 2.65 |  | 1.56 | (0.80-3.05) | 0.189 | 1.53 | (0.79-3.00) | 0.210 |
| p for trend |  |  |  |  |  | 0.277 |  |  | 0.275 |
| **Age ≧60 years old** | (N=5284) | |  |  |  |  |  |  | . |
| Non PPI user | 291 | 12.21 |  | Ref. |  |  | Ref. |  |  |
| PPI user | 315 | 14.95 |  | 1.26 | (1.07-1.48) | 0.005 | 1.26 | (1.07-1.48) | 0.006 |
| Cumulative DDDs |  |  |  |  |  |  |  |  |  |
| <28 | 81 | 14.41 |  | 1.21 | (0.95-1.55) | 0.127 | 1.34 | (1.04-1.71) | 0.021 |
| 28-55 | 80 | 14.75 |  | 1.24 | (0.97-1.59) | 0.090 | 1.27 | (0.99-1.64) | 0.056 |
| 56-93 | 82 | 17.01 |  | 1.44 | (1.12-1.84) | 0.004 | 1.37 | (1.07-1.76) | 0.012 |
| ≧94 | 72 | 13.82 |  | 1.17 | (0.90-1.52) | 0.237 | 1.06 | (0.82-1.38) | 0.650 |
| p for trend |  |  |  |  |  | 0.015 |  |  | 0.089 |
|  | | | | | | | | | |

Adjusted for age, gender, urbanization, all co-morbidities, CCI score, and medication.

PPI: proton pump inhibitor

cHR: crude hazard ratio

aHR: adjusted hazard ratio

DDD: defined daily dose

CCI score: Charlson’s comorbidity index score

**Table B. The risk of all cause dementia between non PPI user and subtype PPI user (N=15,726)**

|  | No. cases | Per 1,000  Person year |  | cHR | (95%CI) | p value | aHR | (95%CI) | p value |
| --- | --- | --- | --- | --- | --- | --- | --- | --- | --- |
| Non PPI user | 341 | 4.54 |  | Ref. |  |  | Ref. |  |  |
| Subtype PPI |  |  |  |  |  |  |  |  |  |
| Omeprazole  (N=4783) | 243 | 5.99 |  | 1.35 | (1.15-1.60) | <0.001 | **1.30** | **(1.09-1.54)** | **0.003** |
| Pantoprazole  (N=1580) | 63 | 4.76 |  | 1.10 | (0.84-1.44) | 0.497 | 1.36 | (0.98-1.89) | 0.067 |
| Lansoprazole  (N=3838) | 161 | 4.98 |  | 1.13 | (0.94-1.37) | 0.193 | 1.20 | (0.98-1.46) | 0.080 |
|  | | | | | | | | | |

Adjusted for age, gender, urbanization, all co-morbidities, CCI score, and medication.

PPI: proton pump inhibitor

cHR: crude hazard ratio

aHR: adjusted hazard ratio

CCI score: Charlson’s comorbidity index score

**Table C. The risk of all-cause dementia stratified by gender, age, all comorbidities, and medication between non–PPI users and PPI users (N = 15 726)**

|  |  | Non PPI user | |  | PPI user | |  | PPI user Verse non PPI user | |
| --- | --- | --- | --- | --- | --- | --- | --- | --- | --- |
|  |  | No.  cases | (%) |  | No.  cases | (%) |  | aHR  (95% CI) | p value |
| Gender |  |  |  |  |  |  |  |  |  |
| Female |  | 160 | (5.0) |  | 169 | (5.2) |  | 1.21(0.97-1.50) | 0.097 |
| Male |  | 181 | (3.9) |  | 197 | (4.3) |  | **1.24(101-1.52)** | **0.040** |
| Age |  |  |  |  |  |  |  |  |  |
| <70 |  | 150 | (2.3) |  | 150 | (2.1) |  | 1.17(0.92-1.48) | 0.202 |
| ≧70 |  | 191 | (15.0) |  | 229 | (17.7) |  | **1.33(1.09-1.61)** | **0.004** |
| Urbanization |  |  |  |  |  |  |  |  |  |
| Urban & suburban |  | 239 | (4.1) |  | 254 | (4.4) |  | 1.29(1.07-1.54) | 0.006 |
| Rural |  | 102 | (5.1) |  | 112 | (5.5) |  | 1.15(0.88-1.52) | 0.309 |
| Comorbidites |  |  |  |  |  |  |  |  |  |
| Diabetics |  |  |  |  |  |  |  |  |  |
| No |  | 271 | (3.9) |  | 304 | (4.3) |  | 1.29(1.09-1.52) | 0.003 |
| Yes |  | 70 | (8.1) |  | 62 | (7.6) |  | 0.99(0.70-1.41) | 0.950 |
| Hyperlipidemia |  |  |  |  |  |  |  |  |  |
| No |  | 275 | (4.0) |  | 290 | (4.2) |  | 1.18(0.99-1.39) | 0.058 |
| Yes |  | 66 | (6.4) |  | 76 | (7.5) |  | 1.49(1.06-2.10) | 0.023 |
| hypertension |  |  |  |  |  |  |  |  |  |
| No |  | 174 | (2.9) |  | 183 | (3.1) |  | 1.23(0.99-1.52) | 0.057 |
| Yes |  | 167 | (8.7) |  | 183 | (9.5) |  | 1.23(0.99-1.52) | 0.057 |
| Depression |  |  |  |  |  |  |  |  |  |
| No |  | 308 | (4.1) |  | 331 | (4.4) |  | 1.20(1.02-1.40) | 0.026 |
| Yes |  | 33 | (8.4) |  | 35 | (9.5) |  | 1.59(0.96-2.65) | 0.074 |
| Ischemic heart disease |  |  |  |  |  |  |  |  |  |
| No |  | 317 | (4.2) |  | 322 | (4.3) |  | 1.16(0.99-1.36) | 0.063 |
| Yes |  | 24 | (7.6) |  | 44 | (14.4) |  | 2.18(1.31-3.63) | 0.003 |
| Cerebral vascular disease |  |  |  |  |  |  |  |  |  |
| No |  | 316 | (4.1) |  | 339 | (4.4) |  | 1.25(1.07-1.46) | 0.005 |
| Yes |  | 25 | (14.5) |  | 27 | (14.7) |  | 1.07(0.61-1.88) | 0.814 |
| Medication |  |  |  |  |  |  |  |  |  |
| Anticoagulant agents |  |  |  |  |  |  |  |  |  |
| No |  | 337 | (4.3) |  | 463 | (4.6) |  | 1.23(1.06-1.43) | 0.008 |
| Yes |  | 4 | (11.4) |  | 3 | (9.1) |  | 6.04(0.27-13.5) | 0.257 |
| Antiplatete agents |  |  |  |  |  |  |  |  |  |
| No |  | 312 | (4.1) |  | 323 | (4.2) |  | 1.20(1.02-1.40) | 0.026 |
| Yes |  | 29 | (12.4) |  | 43 | (18.4) |  | 1.60(0.98-2.60) | 0.062 |
| Antidiabetic agents |  |  |  |  |  |  |  |  |  |
| No |  | 288 | (3.9) |  | 320 | (4.4) |  | 1.28(1.09-1.51) | 0.003 |
| Yes |  | 53 | (9.7) |  | 46 | (8.5) |  | 0.90(0.60-1.35) | 0.606 |
| Antihypertension agents |  |  |  |  |  |  |  |  |  |
| No |  | 215 | (3.4) |  | 211 | (3.3) |  | 1.11(0.92-1.35) | 0.287 |
| Yes |  | 126 | (8.6) |  | 155 | (10.8) |  | 1.46(1.14-1.85) | 0.002 |
| statin |  |  |  |  |  |  |  |  |  |
| No |  | 327 | (4.3) |  | 345 | (4.5) |  | 1.18(1.03-1.40) | 0.022 |
| Yes |  | 14 | (7.6) |  | 21 | (12.3) |  | 2.01(0.92-4.37) | 0.078 |
| NSAIDs |  |  |  |  |  |  |  |  |  |
| No |  | 288 | (4.0) |  | 300 | (4.1) |  | 1.20(1.02-1.41) | 0.029 |
| Yes |  | 53 | (9.0) |  | 66 | (11.0) |  | 1.34(0.94-1.98) | 0.108 |

Adjusted for age, gender, urbanization, all co-morbidities, CCI score, and medication.

PPI: proton pump inhibitor

aHR: adjusted hazard ratio

NSAIDs: non-steroidal anti-inflammatory agents

CCI score: Charlson’s comorbidity index score

**Figure A. All cause dementia by subtype of proton pump inhibitors**
